# Complex Knowledge Base Question Answering for Intelligent Bridge Management Based on Multi-Task Learning and Cross-Task Constraints

**DOI:** 10.3390/e24121805

**Published:** 2022-12-10

**Authors:** Xiaoxia Yang, Jianxi Yang, Ren Li, Hao Li, Hongyi Zhang, Yue Zhang

**Affiliations:** 1 School of Information Science and Engineering, Chongqing Jiaotong University, Chongqing 400074, China; 2 School of Traffic and Transportation, Chongqing Jiaotong University, Chongqing 400074, China

**Keywords:** bridge management, knowledge base question answering, complex question, multi-task learning, cross-task constraints

## Abstract

In the process of bridge management, large amounts of domain information are accumulated, such as basic attributes, structural defects, technical conditions, etc. However, the valuable information is not fully utilized, resulting in insufficient knowledge service in the field of bridge management. To tackle these problems, this paper proposes a complex knowledge base question answering (C-KBQA) framework for intelligent bridge management based on multi-task learning (MTL) and cross-task constraints (CTC). First, with C-KBQA as the main task, part-of-speech (POS) tagging, topic entity extraction (TEE), and question classification (QC) as auxiliary tasks, an MTL framework is built by sharing encoders and parameters, thereby effectively avoiding the error propagation problem of the pipeline model. Second, cross-task semantic constraints are provided for different subtasks via POS embeddings, entity embeddings, and question-type embeddings. Finally, using template matching, relevant query statements are generated and interaction with the knowledge base is established. The experimental results show that the proposed model outperforms compared mainstream models in terms of TEE and QC on bridge management datasets, and its performance in C-KBQA is outstanding.

## 1. Introduction

In contrast to general search engines, question answering (QA) systems can simply and quickly provide queries with accurate and clear answers rather than a series of documents related to natural language questions [1]. The QA system mainly includes three parts: Question analysis, information retrieval, and answer processing. As the main tasks of question parsing, topic entity extraction (TEE) and question classification (QC) play important roles in information retrieval and answer processing [2]. With the continuous development in big data knowledge engineering, data have realized the transformation and upgrading from information to knowledge, and further deepened the research on knowledge base QA (KBQA) [3]. According to the difficulty of the question, it can be mainly divided into simple KBQA and complex KBQA (C-KBQA) [4].

Bridge management is essential to ensure bridges are always in safe condition [5]. In addition to direct measures, such as detection, monitoring, evaluation, maintenance, and reinforcement, bridge management includes some indirect measures, such as data management, emergency plans, and auxiliary decision-making [6]. At present, most of the bridge management data are stored in the form of electronic documents, e.g., inspection reports, which contain large amounts of valuable information to be further excavated and integrated. Taking full advantage of the data can help bridge engineers in analyzing bridge defects and assisting in their judgment and decision-making [7]. Therefore, an intelligent system is urgently needed to realize the structured storage of management data and further realize the effective interaction between bridge engineers and the databases. For the urgent need in intelligent bridge management, the KBQA technology is undoubtedly the best solution.

The KBQA system can be used as a portable intelligent terminal to meet the requirements of fine-grained domain information interaction in bridge management scenarios in real time, thereby supporting knowledge sharing in specific domains and improving work efficiency. For example, bridge engineers can use the KBQA system to query the structural attributes of bridges and the defect information of bridge members, quickly grasp the similar characteristics of bridges in the area, and achieve the purpose of assisting in decision-making. However, the KBQA research that is fully applicable to the bridge engineering field is still in the preliminary exploratory stage [8], and some research in related fields can provide insight [9,10,11], but there are still three main challenges:

(1) Due to the privacy policy, bridge management data have not been fully disclosed, and the industry lacks ready-made knowledge base and question answering corpus, resulting in insufficient data support for research in this field. In addition, important information, such as basic attributes, defect damage, and technical conditions are not fully utilized, leading to insufficient knowledge services in the field of bridge management.
(2) Bridge management information has great domain characteristics in data storage, term description, expression, etc. For example, the topic entity boundary is not clear with a significant amount of specialized words, and the question types are not evenly distributed. Existing methods cannot directly solve these problems.
(3) The bridge management questions are relatively complex, involving multiple-hops, judgment, constraints, numerical calculation, aggregation operation, etc.

**Figure 1:**
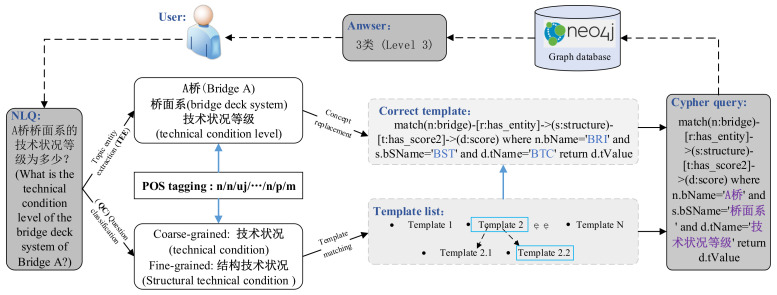
The overall process of C-KBQA for bridge management. For the natural language question (NLQ) given by the user, the purpose of C-KBQA for bridge management is to generate the corresponding Cypher query. Then, the Neo4j graph database is searched to return the answer “3类” (Level 3) to the user. The process of transforming questions into structured queries involves some key technologies, including TEE, POS, and QC.

To solve the above-mentioned problems, this paper proposes a cross-task constraint-enhanced multi-task learning (MTL) framework to solve the C-KBQA of bridge management. Figure 1 shows the overall process of C-KBQA for bridge management.

The proposed framework classifies fine-grained domain C-KBQA as the main task, TEE and QC as two key auxiliary tasks, and part-of-speech (POS) tagging as a secondary auxiliary task. First, we jointly train model embeddings for multiple auxiliary tasks by sharing similar pretrained language encoder models and sharing parameters. Second, POS information, question types, and topic entities are introduced into different subtasks as external knowledge to enhance cross-task semantic constraints. Finally, according to the bridge management domain characteristics of TEE and QC, model improvement and innovation are carried out at the feature fusion layer. The main contributions of this paper are as follows:

(1) We constructed a bridge management domain knowledge base and question answering corpus, realized the integration and utilization of data, and laid the data foundation for the question answering task in this field.
(2) Cross-task constraints (CTC) make the semantics of subtasks interrelated, enrich the expression of contextual semantic features of domain questions, and can effectively solve problems, such as unclear entity boundaries and inaccurate professional vocabulary recognition. MTL strategy can reduce the error propagation of TEE and QC tasks. The template matching method combines semantic analysis and neural network technology to convert natural language questions into answer templates, which can answer more complex questions and maximize the accuracy of QA, meeting the complex application scenarios of bridge management.
(3) This research compensates for the shortage of fine-grained knowledge service in the bridge management field and realizes the fine-grained information interaction between bridge management users and domain knowledge base.

The remainder of the paper is organized as follows. Section 2 provides an overview of related works. Section 3 presents the overall architecture of the proposed model and introduces its key components in detail. Section 4 presents the experimental and evaluation results of the proposed model. Section 5 demonstrates an example of the C-KBQA system platform of bridge management. Finally, Section 6 concludes the paper and outlines future work directions.

## 2. Related Work

With the rapid development in natural language processing (NLP) and neural networks, the research on KBQA has gradually shifted from general domains to specific domains. However, the research related to the field of bridge engineering is still in its infancy. In this section, we reviewed the relevant topics of KBQA.

### 2.1. Complex Knowledge Base Question Answering

Complex questions contain multiple entities and relationships; therefore, C-KBQA involves multiple knowledge triples [12], including operations, such as multi-hop, aggregation, logical operations, and reasoning [13]. At present, the mainstream methods of C-KBQA include semantic parsing, information retrieval, and template matching [14].

Semantic parsing converts natural language questions into logical symbols which can be used on the knowledge base to obtain answers [15]. For example, Sun et al. [16] proposed a multi-strategy method based on semantic parsing that combines sentence-lexical level semantics to represent high-level semantics of complex questions. Zhang et al. [17] proposed a KBQA semantic parsing model based on structural information constraints (SIR). Guo et al. [18] introduced dialogue memory management to manipulate historical entities, predicates, and logical forms, in order to infer the logical form of the current utterance. The method based on semantic parsing can achieve a more interpretable reasoning process by generating a logical form. However, these methods depend heavily on the design of the logical form and the quality of the parser algorithm [19]. It is difficult for existing semantic parsers to cover a variety of complex queries [20].

Information retrieval-based methods extract the relevant information of the questions from the knowledge base, and then rank all the extracted entities and relationships [21]. For instance, Zhou et al. [22] proposed the use of information retrieval to update reasoning instructions in the reasoning stage, in order to improve the reasoning ability of complex problems. Jin et al. [23] decomposed complex questions into multiple triplet patterns, and then retrieved the matching candidate subgraphs from the knowledge base to find answers through a semantic similarity evaluation. Answer ranking is applicable to a small search space [24], while complex questions contain more relationships and subject entities, which increases the difficulty in searching and ranking for candidate answers.

Template matching-based approaches aim to generate structured query statements using predefined templates [25]. In order to perform the question resolution process of C-KBQA, Gomes et al. [26] proposed a hereditary attentive template-based approach for C-KBQA. This method uses the combination of semantic analysis and neural network technology to classify natural language questions into answer templates. However, the limited number of templates and insufficient coverage have become a bottleneck hindering performance improvement [27].

The above-mentioned methods all involve some key subtasks, including TEE, QC, semantic matching, etc. [28]. A popular method of subtask composition is the pipeline model [29]. For example, Chen et al. [30] constructed a KBQA pipeline system to identify the knowledge base relationship corresponding to the question. However, the pipeline model consists of concatenated subtasks, and the prediction results of the previous task will affect the subsequent tasks, thus causing serious error propagation. To solve this problem, MTL has been applied to complex question answering [31,32]. For example, Yang et al. [33] proposed a new multi-tasking and knowledge-enhanced multi-head interactive attention network, which classifies questions as auxiliary tasks and conducts community question answering through multi-tasking learning. In addition, multi-tasking joint learning can enhance the generalization ability of the model [34].

### 2.2. Domain-Specific KBQA Approaches

In recent years, KBQA technologies have begun to be applied in specific fields, e.g., biomedicine [35], finance [36], and education [37]. With the deepening of KBQA research in specific domains, some basic works have been carried out in bridge engineering related fields. For example, Wu et al. [38] discussed the role and challenges of NLP in intelligent construction, providing a reference for the application of NLP technology in the industrial field. In the QA system of building regulations proposed by Zhong et al. [39], the BERT pretraining model is used for feature extraction questions. Li et al. [40] used narrative descriptions in bridge inspection reports as data sources and proposed a data-driven framework to support automatic condition recommendation and real-time quality control. Xia et al. [41] proposed a data-driven bridge condition assessment framework to effectively predict the future condition of bridges. Although the research in related fields can provide reference for the field of bridge management, the existing methods cannot be directly applied to the question answering task of bridge management due to the strong field characteristics.

Furthermore, our previous research laid the foundation for bridge management C-KBQA. For example, the previous work [42] constructed bridge structure and health monitoring ontology using Semantic Web technology, and realized multi-angle fine-grained modeling of bridge structure, SHM system, sensor, and perception data, which lays a foundation for the semantic ontology construction of bridge maintenance. With the in-depth research, Li et al. [43] proposed a dictionary-enhanced machine reading comprehension NER neural model for identifying planes and nested entities from Chinese bridge detection texts. In the previous work [44], a new entity related attention neural network model was proposed for joint extraction of entities and relationships in bridge inspection. The research on domain text information extraction provides technical support for the construction of bridge management knowledge graph. Yang et al. [45] proposed a novel BigKE-based intelligent bridge management and maintenance framework according to the big data knowledge engineering paradigm, pointing out the direction for bridge management knowledge services. However, the above-mentioned work is partial to theoretical research and technical preparation. The core work of bridge management QA task has not been carried out, and the problems, such as insufficient utilization of bridge management data and insufficient domain knowledge service, have not been effectively solved.

### 2.3. Gaps and Challenges 

Research status shows that the existing methods cannot be directly applied to the field of bridge management, and bridge management C-KBQA faces many challenges. Therefore, combined with the existing theories and methods, as well as the characteristics of the field, a reasonable solution for bridge management C-KBQA is briefly presented.

(1) The bridge management process is concerned with numerical values, such as technical condition level and score, as well as textual information, such as structural defect and maintenance advice. Therefore, the Neo4j attribute graph structure can be used for storing bridge management knowledge. In addition, the question types in the field of bridge management are relatively fixed; therefore, template matching can give full scope to its advantages, which can better ensure the QA effect in the practical application scenarios of bridge management.
(2) Bridge management questions often contain multiple topic entities with ill-defined boundaries. For example, the question “A桥桥面系的技术状况等级是多少？” (“What is the technical condition level of the bridge deck system of Bridge A?”) contains two topic entities “A桥” (“Bridge A”) and “桥面系” (“Deck System”). The same character “桥” (“bridge”) exists between topic entities, but without any separators. In addition, the short text of bridge management questions lacks contextual semantics, which increases the difficulty in professional vocabulary recognition. Moreover, bridge management questions can be classified according to two levels: Coarse-grained and fine-grained. For example, the coarse-grained type for the question “A桥和B桥有哪些共同缺陷？” (“What common defects do Bridge A and Bridge B have?”) is “损伤缺陷” (“damage defects”), and its fine-grained type is “共同缺陷” (“common defects”). Therefore, it is necessary to clarify the entity boundary and assist in the fine classification of questions through POS tagging. To improve the contextual comprehension ability of questions, the semantic correlation between subtasks should be strengthened to form CTC. (Due to the privacy property of bridge management information, letters are used rather than real bridge names in this paper).
(3) There is no strict execution order between the TEE and QC tasks. Therefore, the multiple subtasks of bridge management C-KBQA can be jointly trained to avoid error propagation and save computational resources.

## 3. Methodology

**Figure 2:**
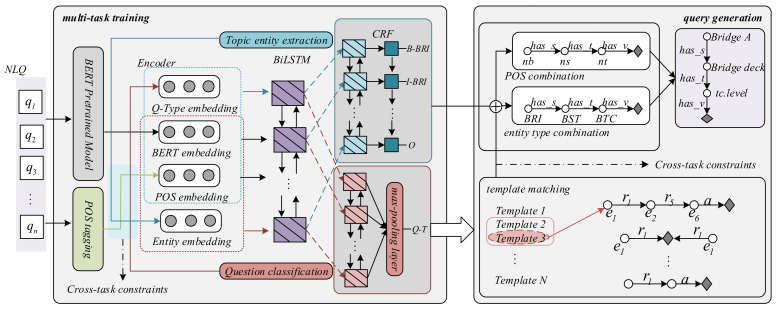
Model architecture of C-KBQA for bridge management. The multi-task learning module (**left part**) consists of four key components: Coding layer, part-of-speech (POS) tagging, topic entity extraction (TEE), and question classification (QC). POS is used for obtaining domain POS features. The encoder layer is composed of question-type embeddings, BERT embeddings, POS embeddings, and entity embeddings, and forms cross-task semantic constraints. Shared BiLSTM is used for extracting features; CRF and maximum pooling are used for decoding TEE and QC tasks. The query generation module (**right part**) matches the template based on the QC result, replaces the concepts in the template based on the TEE result, and aligns with the POS.

Aiming at the domain characteristics of the bridge management C-KBQA, we propose an MTL model that joints POS tagging, TEE, and QC. First, we analyze each subtask module and then elaborate on the constraint strategy. As shown in Figure 2, the model adopts BERT as a shared character-grained embedding model for TEE and QC. In order to make full use of the correlation information between different tasks and achieve CTC, question-type embeddings, BERT character embeddings, and POS embeddings are used as joint encodings for TEE. Similarly, BERT character embeddings, POS embeddings, and entity embeddings are used as joint encodings for QC. Bi-directional long short-term memory (BiLSTM) network is shared for feature extraction, conditional random field (CRF) is used as a decoder for TEE, and question types are obtained by max pooling. According to the output of the multi-task module, the template is matched and the semantic map is constructed. Under the constraints of POS and entity type combinations, the query path is locked, and finally a query is generated according to the extracted topic entities. In contrast to the end-to-end deep learning framework, the proposed model framework is a combination of semantic analysis and deep learning. For specific domain question answering tasks with relatively fixed question types, template matching can ensure the interpretability of the model and maximize the accuracy of question answering. At the same time, it can also avoid the problems of over fitting, gradient explosion, and waste of computing power caused by deep neural network stacking. Therefore, the template matching question answering method is more suitable for the actual application scenario of bridge management.

### 3.1. Encoder

The model classifies the complex questions of bridge management as input, and obtains BERT embeddings, POS embeddings, entity embeddings, and question-type embeddings from character-level, POS-level, word-level, and sentence-level, respectively.

BERT contains three embeddings: Token embeddings, segment embeddings, and position embeddings. The three embeddings are added element-wise to obtain the synthesized embedding. The BERT model encodes embedded features through a transformer component with a multi-head attention mechanism. The encoded character-level distributed representation is expressed as follows:

(1) $$e_{bert}=M_{bert}\left(x_{c}\right)$$

where $x_{c}$ represents the text embedding of the characters in the question, $e_{bert}$ represents the character-level feature embedding encoded by the BERT model, and $M_{bert}$ represents the BERT model encoding component.

POS is the basic grammatical attribute of vocabulary, which contains rich semantic information. The domain POS embedding lookup table $M_{pos}$ is trained by one-hot encoding, and the POS embedding is expressed as follows:

(2) $$M_{pos}=One\_Hot\left\{p_{1},p_{2},\ldots ,p_{n}\right\}\;e_{pos}=M_{pos}\left(p\right)$$

where $\left\{p_{1},p_{2},\ldots ,p_{n}\right\}$ represent the POS set, and $e_{pos}$ represents the encoding of the POS in the lookup table.

The entity embedding matrix uses the domain specialized vocabulary as the database, and uses Glove for entity embedding. The topic entity embedding will be used as the embedding constraint for the QC task, which is expressed as follows:

(3) $$e_{te}=M_{te}\left(e_{t}\right)$$

Similarly, the question-type embedding is also obtained by one-hot encoding that will be used as the embedding constraint of the TEE task, which is expressed as follows:

(4) $$e_{qt}=M_{qt}\left(t_{q}\right)$$

### 3.2. Multi-Task Learning

The MTL module includes three key subtasks: POS tagging, TEE, and QC. Character-grained embedding of subtasks and feature extraction are achieved by sharing the BERT encoder and BiLSTM feature extractor.

**Figure 3:**
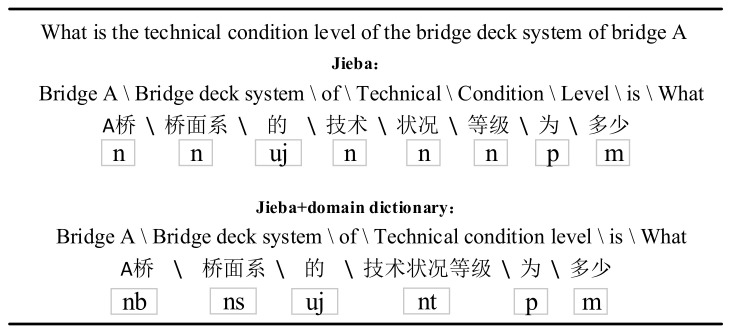
Comparative example of POS tagging in bridge management questions. The former is the POS tagging result of Jieba tool, and the latter is the POS tagging result after adding the domain dictionary.

***Part-of-speech tagging***: POS tagging includes word segmentation and POS judgment. POS tagging can effectively distinguish entity boundaries. The combination of different POS in questions will also affect the judgment of QC. Formally, the question Q can be divided into multiple word combinations {$w_{1},\;w_{2},\ldots ,\;w_{n}$} and then given the POS list {$pos_{1},\;pos_{2},\ldots ,\;pos_{n}$} in the word combination. POS tagging is used for generating POS embeddings and providing POS combination constraints during the query generation stage. As shown in Table 1, we build a professional vocabulary library for bridge management and define the vocabulary attributes of domain vocabulary to improve the performance of Jieba. Figure 3 shows the two POS tagging results of bridge management questions by Jieba tool. The former lacks the domain POS, resulting in incorrect word segmentation. Clearly, the latter POS tagging results contain more domain semantic information.

**Figure 4:**
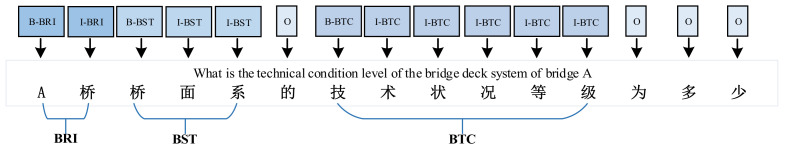
Example of topic entity extraction. The topic entities of the question are “A桥” (“Bridge A”, “桥面系” (“Deck System”), and “技术状况等级” (“Technical conditions level”). The corresponding entity labels are “BRI”, “BST”, and “BTC”, respectively.

***Topic entity extraction***: The primary purpose of TEE is to extract key substrings $e_{t}=\left(c_{1}^{t},c_{2}^{t},\ldots ,c_{m}^{t}\right)$ from question string $Q=\left(c_{1}^{q},c_{2}^{q},\ldots ,c_{n}^{q}\right)$, where m≤n. Then, determine the entity type $t_{e}$ of $e_{t}$, where $t_{e}$ belongs to the list of entity labels {BRI, BST, BSE, BSL, BSD, BTC}. An example of TEE is shown in Figure 4. The meaning and examples of entity labels are shown in Table 1.

The features of question-type embeddings, BERT embeddings, and POS embeddings are fused to obtain the final encoding for TEE task. The embedding vector is expressed as follows:

(5) $$E_{te}=\left\{e_{qt};e_{bert};e_{pos}\right\}$$

Taking $E_{te}$ as the input of BiLSTM to capture long-range dependencies and extract features, the BiLSTM model consists of forget gate $f_{t}$, input gate $i_{t}$, temporary cell state ${\tilde{C}}_{t}$, cell state $C_{t}$, output gate $o_{t}$, and bidirectional hidden state $\left[\vec{h};\overset{\leftarrow }{h}\right]$. The status at each time step is expressed as follows:

(6) $$f_{t}=\sigma \left(W_{f}\cdot \left[{\vec{h}}_{t-1},x_{t}\right]+b_{f}\right)\;i_{t}=\sigma \left(W_{i}\cdot \left[{\vec{h}}_{t-1},x_{t}\right]+b_{i}\right)\;{\tilde{C}}_{t}=tanh\left(W_{C}\cdot \left[{\vec{h}}_{t-1},x_{t}\right]+b_{C}\right)\;C_{t}=f_{t}*C_{t-1}+i_{t}*{\tilde{C}}_{t}\;o_{t}=\sigma \left(W_{o}\cdot \left[{\vec{h}}_{t-1},x_{t}\right]+b_{o}\right)\;{\vec{h}}_{t}=o_{t}*tanh\left(C_{t}\right)$$

where $W_{f}$, $W_{i}$, $W_{C}$, $W_{o}$, $b_{f}$, $b_{i}$, $b_{C}$, and $b_{o}$ are the weight parameters that need to be learned during the training process, and $x_{t}$ is the question embedding word vector at the current moment. The forward hidden vectors $\left\{{\vec{h}}_{0},{\vec{h}}_{1},\ldots ,{\vec{h}}_{n-1}\right\}$ and backward hidden vectors $\left\{{\overset{\leftarrow }{h}}_{n-1},{\overset{\leftarrow }{h}}_{n-2},\ldots ,{\overset{\leftarrow }{h}}_{0}\right\}$ are spliced to obtain the final hidden layer state as follows:

(7) $$h=\left[\vec{h};\overset{\leftarrow }{h}\right]=\left\{\left[{\vec{h}}_{0};{\overset{\leftarrow }{h}}_{n-1}\right],\left[{\vec{h}}_{1};{\overset{\leftarrow }{h}}_{n-2}\right],\ldots ,\left[{\overset{\leftarrow }{h}}_{n-1};{\overset{\leftarrow }{h}}_{0}\right]\right\}$$

The CRF layer classifies the emission score of BiLSTM as input and outputs the maximum possible predicted annotation sequence that meets the annotation transfer constraints. The model loss function is defined as follows:

(8) $$P\left(y|x\right)=\frac{\exp\left(score\left(x,y\right)\right)}{\sum_{\tilde{y}\in Y_{X}}\exp\left(score\left(x,\tilde{y}\right)\right)}\;score\left(x,y\right)=\overset{n}{\underset{i=0}{\sum }}P_{i,y_{i}}+\overset{n}{\underset{i=0}{\sum }}A_{y_{i},y_{i+1}}$$

where $P_{i,y_{i}}$ and $A_{y_{i},y_{i+1}}$ represent the emission score and transition score of $y_{i}$ in the labeling sequence y, respectively. The maximum log-likelihood function during model training is expressed as follows:

(9) $$log\left(P\left(y|x\right)\right)=score\left(x,y\right)-log(\underset{\tilde{y}\in Y_{X}}{\sum }\exp\left(score\left(x,\tilde{y}\right)\right))$$

***Question classification***: QC aims to determine the type $t_{q}$ of the question Q and establish the mapping relation $f\left(Q\right)=t_{q}$. For example, the coarse-grained type of the question in Figure 4 is “技术状况” (“technical condition”) and the fine-grained type is “结构技术状况” (“structural technical condition”). The features of BERT embeddings, POS embeddings, and entity embeddings are fused to obtain the final encoding for the QC task. The embedding vector is expressed as follows:

(10) $$E_{qt}=\left\{e_{bert};e_{pos};e_{te}\right\}$$

Taking $E_{qt}$ as the input of the text recurrent convolutional neural network (TextRCNN), the recurrent neural network (RNN) adopts BiLSTM to guarantee model similarity for MTL. BiLSTM is used for capturing the semantic features of the word itself and the left and right context in the question. The left and right context vectors are calculated as follows:

(11) $$e_{l}\left(w_{i}\right)=\sigma \left(W^{\left(l\right)}e_{l}\left(w_{i-1}\right)+W^{\left(sl\right)}E_{qt}\left(w_{i-1}\right)\right)\;e_{r}\left(w_{i}\right)=\sigma \left(W^{\left(r\right)}e_{r}\left(w_{i+1}\right)+W^{\left(sr\right)}E_{qt}\left(w_{i+1}\right)\right)$$

where $W^{\left(l\right)}$, $W^{\left(r\right)}$, $W^{\left(sl\right)}$, and $W^{\left(sr\right)}$ are the weight parameters that need to be learned, $e_{l}\left(w_{i}\right)$ and $e_{l}\left(w_{i-1}\right)$ are the left context embeddings of the current word and the previous word, respectively, $e_{r}\left(w_{i}\right)$ and $e_{r}\left(w_{i+1}\right)$ are the right context embeddings of the current word and the previous word, respectively, and σ is the sigmoid activation function. The vector representation of the current word after feature fusion is expressed as follows:

(12) $$x_{i}=\left[e_{l}\left(w_{i}\right);E_{qc}\left(w_{i}\right);e_{r}\left(w_{i}\right)\right]$$

Perform linear transformation and tanh activation on $x_{i}$ to obtain a latent semantic vector $y_{i}^{1}$. After maximum pooling, the maximum value $y_{i}^{2}$ of the vector elements in $y_{i}^{1}$ is obtained. The formula is expressed as follows:

(13) $$y_{i}^{1}=tanh\left(W_{1}x_{i}+b_{1}\right)\;y_{i}^{2}=\begin{array}{c} n\\ max\\ i=1 \end{array}y_{i}^{1}$$

where $W_{1}$ and $b_{1}$ are hyperparameters that need to be learned during training, and the model output and probability are expressed as follows:

(14) $$y_{i}^{3}=W_{2}y_{i}^{2}+b_{2}\;p_{i}=\frac{\exp\left(y_{i}^{3}\right)}{\sum_{k=1}^{n}\exp\left(y_{i}^{3}\right)}$$

***Objective function***: The main training tasks of the MTL module are TEE and QC. Two key subtasks are jointly trained. The objective functions are expressed as follows:

(15) $$L=\alpha L_{tee}+\beta L_{qc}$$

where $L_{tee}$ and $L_{qc}$ are the loss function for TEE and QC, α and β are the hyperparameters that need to be learned to control the importance of each subtask.

### 3.3. Cross-Task Constraints

The proposed model adopts different CTC in the MTL and query generation stages to improve the model performance. Semantic constraints are used in the MTL stage to enrich the embedded features of subtasks. Conditional constraints are used in the query generation stage to further prune query paths. The key to query generation is template matching and concept replacement. The question template is matched by QC, and the concepts in the template are replaced by TEE, thereby transforming the question into a structured query. Formally, there is a mapping relationship $g\left(t_{q}\right)=T$ between question types $t_{q}$ and query templates T. Templates are structured representations at the conceptual level, replacing conceptual descriptions in T with instances of topic entities $e_{t}$ to obtain the Cypher query $q_{c}$. Semantic constraints are embodied in the encoding layer of the model, while conditional constraints include three steps and two limitations.

***Step 1***: Narrow down templates based on coarse-grained question types and determine query templates based on fine-grained question types.

***Limitation 1***: $t_{i}\in T_{range}$, where $t_{i}$ indicates the matched correct template and $T_{range}$ indicates the locked template range.

***Step 2***: Add potential constraints to query templates and prune query paths based on POS and entity-type combinations. Correct the TEE result according to POS information.

***Limitation 2***: Ensure the POS combination is consistent with the entity-type combination (entity and POS tags are detailed in Table 1).

***Step 3***: The Cypher query is generated by replacing the concept description in the query template with an instance of the topic entity.

## 4. Experiments

Utilizing the constructed knowledge base and C-KBQA corpus for bridge management as domain data sources, we evaluate the performance of the proposed model and conduct experimental comparisons.

### 4.1. Neo4j Knowledge Base

**Figure 5:**
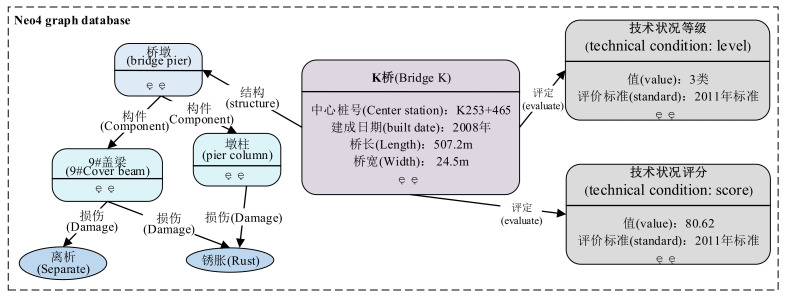
The example of Neo4j knowledge base for bridge management. Neo4j is an attribute graph. The entity node contains some basic attributes. For example, the value of the attribute “中心桩号” (“Center Station”) of the bridge entity “K桥” (“Bridge K”) is “K253+465”. Different entities are connected by relational edges to form a graph structure. For example, there is a “结构” (“Structure”) relationship between “K桥” (“Bridge K”) and “桥墩” (“Bridge Pier”).

Combined with the practical application scenarios of bridge management and the characteristics of domain data, the Neo4j graph database is used for storing bridge management knowledge. Data are obtained from an unstructured text, semi-structured tables, and relational data. Based on the previous research work [8], the extracted triple knowledge is stored in the Neo4j graph database. As shown in Figure 5, the bridge maintenance knowledge base is composed of various types of bridge entities and relation edges, and the entity nodes contain basic attributes. The constructed bridge maintenance knowledge base contains 9352 entities, 19,556 relationships, and 5674 attribute values.

### 4.2. Experimental Dataset

**Figure 6:**
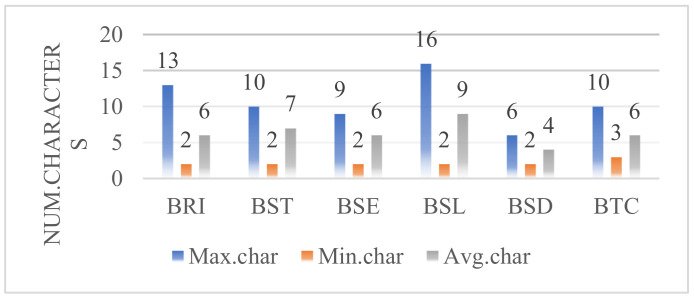
The number of characters for various entities. Max.char, Min.char, and Avg.char, respectively represent the maximum, minimum, and average characters of different types of entities.

Aiming at the multi-task strategy of domain C-KBQA, the QA, TEE, and QC datasets are constructed, respectively. The annotated corpus consists of 26,798 questions with information about 126 bridges, which contain 513,631 characters. The corpus is divided into training, validation, and test sets according to 8:1:1. For the TEE task, Table 2 shows the distribution of entities in its training, validation, and test sets. Figure 6 shows the number of characters for various entities.

The bridge management questions are divided into coarse-grained and fine-grained. There are three types of coarse-grained questions and ten types of fine-grained questions. Table 3 shows the number of various bridge management questions.

The 2680 questions in the test set were selected to construct QA pairs to verify the C-KBQA effect of bridge management. Complex questions are often characterized by multiple-hops, numerical calculations, aggregation, etc. Combining the question characteristics in the bridge management domain, Table 4 lists some examples of complexity in bridge management QA pairs. In the table, the multi-hop questions indicate that the questions involve multiple knowledge triples. Numerical calculation questions are mainly in regrad to the types of defects and the number of components. The questions of judgment, constraint, and aggregation operation have certain inference properties, which are difficult questions in bridge management C-KBQA.

### 4.3. Baselines and Configurations

The precision, recall, and *F*1 score are used for evaluating the performance of TEE tasks. Accuracy and *F*1 score are used as evaluation metrics for QC tasks. The calculation formulas of these evaluation metrics are expressed as follows:

(16) $$Accuary=\left(TN+TP\right)/\left(TN+TP+FN+FP\right)\;Precision=TP/\left(TP+FP\right)\;Recall=TP/\left(TP+FN\right)\;F1=2*Precision*Recall/\left(Precision+Recall\right)$$

where TP is true positive, TN is true negative, FP is false positive, and FN is false negative. Accuary represents the ratio of the number of correctly classified samples to the total number of samples. Precision represents the ratio of the number of correctly retrieved samples to the total number of retrieved samples. Recall represents the ratio of the number of samples that were correctly retrieved to the number of samples that should have been retrieved. F1 is the weighted harmonic mean of Precision and Recall. The baseline models for the TEE task include CNN-CRF, BiLSTM-CRF, and BERT-CRF. The baseline models for the QC task include TextRNN_Att, TextRCNN, ERNIE, BERT, BERT_CNN, and BERT_RNN.

All experiments were conducted on a server with Intel Xeon Gold 6338 CPU, NVIDIA A40 GPU, 32 GB DDR3 RAM, and 512 GB disk space. For the MTL of C-KBQA, the parameter settings on the two key subtasks of TEE and QC are shown in Table 5. The parameter settings of the pretrained model mainly use the native embedding dimension and hyperparameters of Bert_base_Chinese.

### 4.4. Experimental Results

The performance comparison of TEE task is shown in Table 6, BiLSTM outperforms CNN, and its *F*1 score is improved by 1.01%. The BERT pretraining model is effective, and its *F*1 score is 2.04% higher than BiLSTM. Based on BERT, our proposed model improves the domain adaptability and achieves the expected effect. Its *F*1 score is 94.76%, which is better than the above baselines.

The model performance comparison for the QC task is shown in Table 7. For the text classification series model, TextRCNN performed best with an *F*1 score of 96.79%. The *F*1 score of the BERT model is 1.87% higher than the TextRCNN model. The BERT series model is significantly better than the text classification model. Our proposed model introduces MTL and CTC on BERT, and the *F*1 score is 98.84%, which has well model performance.

For the bridge management C-KBQA task, this paper conducts question answering experiments on 2680 complex questions in the test set. The trained multi-task model is used for QC and TEE, and structured queries are generated according to template matching principles and concept replacement rules. A total of 10 overall experiments were conducted on 2680 questions, and the average test scores and standard deviations of the 10 results were used as comprehensive evaluation indicators. The experimental results shown in Table 8 demonstrate that the model effect of basic information question and technical condition question is remarkable, and the comprehensive *F*1 score of bridge management C-KBQA also reaches more than 90%, which initially meets the needs of specific scenarios. However, the accuracy of damage defects needs to be improved, and it will be the work of continuous optimization and improvement in this paper. This is due to the fact that the damage defect question involves multi-hop retrieval calculation, and the question scenario is more complicated.

In addition, we compare the proposed model framework with the latest general framework, as shown in Table 9. Comparison models are some general domain models based on information retrieval ideas. They are mainly encoded by pretraining models, and then some deep neural networks can be accessed for feature extraction. Finally, answers are matched by semantic similarity calculation. However, the comparison model does not improve the model according to the characteristics of the domain, nor does it introduce the domain rules, which is quite different from the template matching method. The experimental results show that the indexes of the proposed model are clearly better than the comparison models, which proves the superior performance of the proposed model based on template matching in the C-KBQA task of bridge management. It is further proved that the existing deep learning techniques cannot be directly applied to the C-KBQA scene with the knowledge inference property in a specific domain.

### 4.5. Ablation Study

Furthermore, we evaluate the MTL model performance and cross-task constraint effect of bridge management C-KBQA. As shown in Table 10, the single-task experiments are equivalent to the use of the BERT-BiLSTM-CRF and BERT-RCNN models independently for TEE and QC, respectively. Subsequently, the semantic embedding constraints of each subtask are sequentially added to other tasks, and the BERT and model parameters are shared, thereby proving the effectiveness of each key component in the model. The experimental results show that the performance of the MTL model is significantly better than the single-task model.

## 5. System Prototype

**Figure 7:**
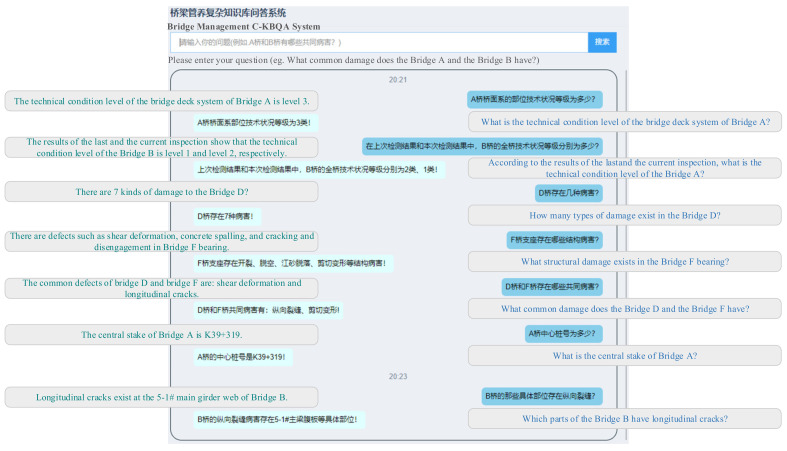
C-KBQA prototype system for bridge management. The system takes the proposed model as the back-end technical support. The right part consists of the natural language questions in the field of bridge management input by domain users, and the left part consists of the answers returned by the system after analysis and processing. In addition, the answer is in a natural language and contains the context, which makes the human-computer interaction more friendly.

To evaluate the effect of the proposed C-KBQA method in bridge management scenarios, we developed a bridge management C-KBQA prototype system. As shown in Figure 7, when logging in to the system interface, bridge management users can query bridge management-related information in natural language, and the system will directly return the processed answer. The question answer module includes the retrieval function of fact triple knowledge, the enumeration function of various bridge defects, the inference function of bridge common defects, and the calculation function of bridge defect types. In this way, information retrieval, structural state evolution trend, technical condition assessment, and damage safety warning among multiple bridges with similar structure types and similar operating environments are realized, and the bridge management unit can make corresponding decisions.

## 6. Conclusions

The informatization in the field of bridge engineering has developed rapidly. Large amounts of data have been accumulated in the process of bridge management, which has laid the foundation for the digital development in bridge management. This paper proposes an MTL framework based on CTC, with POS tagging, TEE, and QC as auxiliary tasks, to improve the performance of bridge management C-KBQA task by sharing BERT and jointly setting model parameters. The proposed model combines BERT embeddings, POS embeddings, entity embeddings, and question-type embeddings to enhance the contextual representation of short-text questions and achieve cross-task semantic constraints. Moreover, the proposed model is experimentally evaluated on the bridge management dataset and compared with the baseline. The results demonstrate that the model outperforms the baseline model on both TEE and QC tasks. Furthermore, the proposed MTL framework achieves good performance in solving the main task of bridge management C-KBQA. The research provides knowledge-based services and intelligent decisions for bridge management users, which has important significance and far-reaching influence on accelerating the information construction of bridge engineering, and provides a new application scenario for the interdisciplinary research of computer science and bridge engineering.

However, the proposed model has certain limitations. Some future work should be carried out as follows:

(1) The template still needs to be designed manually and is closely related to the storage form of the knowledge base; therefore, it cannot be flexibly improved. As a result, the automatic generation of query templates will be the focus of future research. The following work will carry out syntactic dependency analysis and part of speech analysis on natural language questions to obtain the core sentence pattern of the questions. The QA pairs are used as training data, and feature extraction is carried out through the deep neural network to automatically construct question templates.
(2) The bridge management knowledge base contains large numbers of entities and relationships; therefore, the follow-up work can build a domain knowledge graph embedding model to integrate prior knowledge into the model in advance. In order to retain the structural and semantic features between entities and relations at the same time, the graph neural network and attention mechanism will be combined to generate the knowledge graph embedding to capture the deep interaction information between entities and relationships.
(3) With the advancement in deep learning, end-to-end C-KBQA models have been extensively studied in the general domain; therefore, researching C-KBQA end-to-end models for bridge management is another important and challenging task. The future work will be based on the semantic support of knowledge graph embedding, and automatically construct structured queries through the automatic generation of templates, in an attempt to obtain an automatic question answering solution for complex knowledge bases with enhanced domain knowledge semantics.

## Figures and Tables

**Table 1:** The examples of entity and POS labels. There are six types of entity and POS labels. Although the label symbols are different, the corresponding entity instances are the same.

Entity Name	Entity Labels	POS Labels	Vocabulary Examples
Bridge	BRI	nb	Bridge A (A桥)
Bridge Structure	BST	ns	Bridge deck system (桥面系)
Structural Element	BSE	ne	Left web plate (左腹板)
Structural Location	BSL	nl	Within 2 m (2米范围内)
Structural Defect	BSD	nd	Mesh crack (网状裂缝)
Technical Condition	BTC	nt	Technical condition (技术状况)

**Table 2:** The number of entities in the training, validation, and test sets.

Entity Types	Training Set	Validation Set	Test Set	Total
BRI	21,438	2680	2731	26,849
BST	14,432	1961	2248	18,641
BSE	2073	301	198	2572
BSL	1483	189	257	1929
BSD	5124	682	776	6582
BTC	10,940	1504	1325	13,769

**Table 3:** Number of different types of bridge management questions.

Coarse-Grained	Fine-Grained	Number	Total
基础信息 (Basic information)	桥梁信息 (Bridge information)	3018	6283
	结构信息 (Structural information)	3265	
损伤缺陷 (Damage defect)	桥梁缺损 (Bridge defect)	126	11,400
	结构缺损 (Structural damage)	4047	
	缺损描述 (Defect description)	3970	
	缺损属性 (Defect properties)	673	
	缺损位置 (Defect location)	1056	
	共同缺损 (Common defect)	1528	
技术状况 (Technical condition)	桥梁技术状况 (Bridge technical condition)	504	9115
	结构技术状况 (Structural technical condition)	8611	

**Table 4:** The examples of complexity in bridge management QA pairs. Bridge management questions mainly include the following five complex types: Multi-hops, numerical calculation, judgment, constraint, and aggregation operation. Examples of QA pairs for each complex type are provided in the table, where Q stands for “question” and A stands for “answer”.

Complex Types	QA Pairs Examples
Multiple-hops	Q: What is the technical condition level of the bridge deck system of Bridge A? (A桥桥面系的技术状况等级为多少?) A: The technical condition level of the bridge deck system of Bridge A is level 3. (A桥桥面系的技术状况等级为3类.)
Numerical calculation	Q: How many defects exist in Bridge D? (D桥存在几种缺陷?) A: There are seven defects in Bridge D. (D桥有7种缺陷.)
Judgment	Q: Are Bridge B and Bridge C the same technical condition level? (A桥和B桥的技术状况等级一样吗?) A: Bridge B and Bridge C are the same technical condition level. (A桥和B桥的技术状况等级一样.)
Constraint	Q: What are the bridges with technical condition level 1? (技术状况等级为1类的桥有哪些?) A: The bridges with technical condition level 1 are Bridge E, Bridge J, and Bridge I. (技术状况等级为1类的桥有E桥, J桥和I桥.)
Aggregation operations	Q: What defects are common to Bridge D and Bridge F? (D桥和F桥存在哪些共同缺损?) A: The common defects are shear deformation and longitudinal cracks. (D桥和F桥的共同缺损有剪切变形和纵向裂缝.)

**Table 5:** Parameter settings for TEE and QC.

Parameters	TEE Task	QC Task
Pretrained model	Bert_base_Chinese	Bert_base_Chinese
Optimizer	AdamW	AdamW
Batch size	64	64
Learning rate	0.00005	0.00005
Word vector size	100	100
BiLSTM_hidden_size	128	128
Epoches	30	15

**Table 6:** The comparative experimental results of TEE task. The bold numbers represent the best experimental results.

Models	Precision (%)	Recall (%)	F1 (%)
CNN-CRF	90.32	91.47	90.89
BiLSTM-CRF	92.51	91.29	91.90
BERT-CRF	**94.25**	93.63	93.94
Our model	93.86	**95.68**	**94.76**

**Table 7:** The comparative experimental results of QC task. The bold numbers represent the best experimental results.

Models	Accuracy (%)	Precision (%)	Recall (%)	F1 (%)
TextRNN_Att	98.02	97.97	94.81	95.87
TextRCNN	98.26	96.67	96.94	96.79
ERNIE	99.14	98.35	98.71	98.51
BERT	99.18	98.40	98.94	98.66
BERT_CNN	99.10	98.33	98.87	98.59
BERT_RNN	99.12	98.19	**99.03**	98.57
Our model	**99.26**	**98.68**	99.01	**98.84**

**Table 8:** The experimental results of bridge management C-KBQA. The symbol “±” indicates that the results of each experiment fluctuate within the positive and negative range.

Question Types	Precision (%)	Recall (%)	F1 (%)
Basic information	92.32 ± 0.02	87.9 ± 0.03	90.06 ± 0.03
Damage defect	82.57 ± 0.03	78.89 ± 0.03	80.69 ± 0.05
Technical condition	95.24 ± 0.02	92.65 ± 0.01	93.93 ± 0.02
Overall evaluation	91.17 ± 0.02	88.93 ± 0.01	90.04 ± 0.02

**Table 9:** Comparison of C-KBQA models for bridge management. “Similarity” indicates semantic similarity calculation.

Models	Precision (%)	Recall (%)	F1 (%)
BERT-Similarity	67.93	66.25	67.08
BERT-BiLSTM-Similarity	72.31	70.89	71.59
BERT-BiLSTM-Attention	75.58	75.04	75.31
Our Model	91.17	88.93	90.04

**Table 10:** Ablation results of the proposed model. The bold numbers represent the best experimental results.

Task Types	TEE			QC			C-KBQA
	P (%)	R (%)	*F*1 (%)	P (%)	R (%)	*F*1 (%)	*F*1 (%)
Single-Task	94.65	93.32	93.98	98.21	98.89	98.54	88.32
+POS emb	94.79	93.23	94.00	98.16	98.97	98.66	89.43
+Entity.emb	/	/	/	98.48	**99.07**	98.77	/
+Q-Type.emb	**94.95**	93.87	94.41	/	/	/	/
Our model	94.89	**94.64**	**94.76**	**98.68**	99.01	**98.84**	**90.04**
